# Role of PET imaging in evaluating right ventricular function and prognosis in pulmonary hypertension

**DOI:** 10.1016/j.clinsp.2026.101101

**Published:** 2026-09-07

**Authors:** Samara Pinto, Giordana Salvi de Souza, Cristina Maria Moriguchi Jeckel, Luiz Carlos Bodanese, Carlos A. Buchpiguel, Rogério Souza, Ana Maria Marques da Silva

**Affiliations:** a Escola de Medicina, Pontifícia Universidade Católica do Rio Grande do Sul, PUCRS, Rio Grande do Sul, Porto Alegre, Brazil; b Escola de Ciências da Saúde e da Vida, Pontifícia Universidade Católica do Rio Grande do Sul, PUCRS, Porto Alegre, RS, Brazil; c Faculdade de Medicina, Universidade de São Paulo, USP, São Paulo, SP, Brazil

**Keywords:** Pulmonary hypertension, Right ventricular function, Positron emission tomography, Quantification, Molecular imaging

## Abstract

**Aim:**

Pulmonary Hypertension (PH) is a complex pathophysiological condition associated with high morbidity and mortality. Regardless of etiology, Right Ventricular (RV) dysfunction remains a major determinant of prognosis. This integrative review summarizes the current evidence on the role of Positron Emission Tomography (PET) imaging in evaluating disease severity and exploring associations with clinical outcomes in PH, emphasizing how different tracers capture the biological processes underlying RV dysfunction.

**Materials and methods:**

A systematic literature search was performed using PubMed, EMBASE, Scopus, and Web of Science, identifying 35 eligible studies. The review discusses PET tracers, technical aspects of image processing and quantification techniques, and metrics correlated with physiological measures, highlighting their diagnostic and prognostic implications.

**Results:**

PET tracers used for RV assessment in PH include [^13^N]NH_3_, [^11^C]acetate, [¹⁸F]FDG, [^68^Ga]FAPI, and [¹⁸F]LMI1195, with [¹⁸F]FDG being the most widely employed. Quantification predominantly relies on the standardized uptake value (SUV) and kinetic modeling parameters. Common metrics include SUV_mean_, SUV_max_, and the RV/Left Ventricle (LV) SUV ratio (SUV_RV/LV_), which improves inter-patient comparability. However, variability in acquisition and analysis methods limits cross-study comparability. Some studies report SUV_RV/LV_ thresholds above 1.0 as predictors of adverse outcomes, including increased mortality, greater RV dysfunction, and disease severity.

**Conclusion:**

PET imaging represents a valuable, noninvasive modality for evaluating RV function and prognosis in PH. Elevated SUV and SUV_RV/LV_ have been reported to be associated with adverse outcomes in several studies. However, methodological heterogeneity limits cross-study comparability. Future studies should aim to standardize acquisition and quantification protocols and include larger prospective studies.

## Highlights

• Comprehensive synthesis of PET imaging for RV assessment in PH.
• Multi-tracer PET imaging captures distinct RV pathophysiological domains.
• Quantitative PET metrics are linked to disease severity and prognosis.
• Methodological heterogeneity limits inter-study comparability and clinical translation.
• Standardization is essential for clinical translation and prognostic use of PET in PH.

## Introduction

**Fig. 1:**
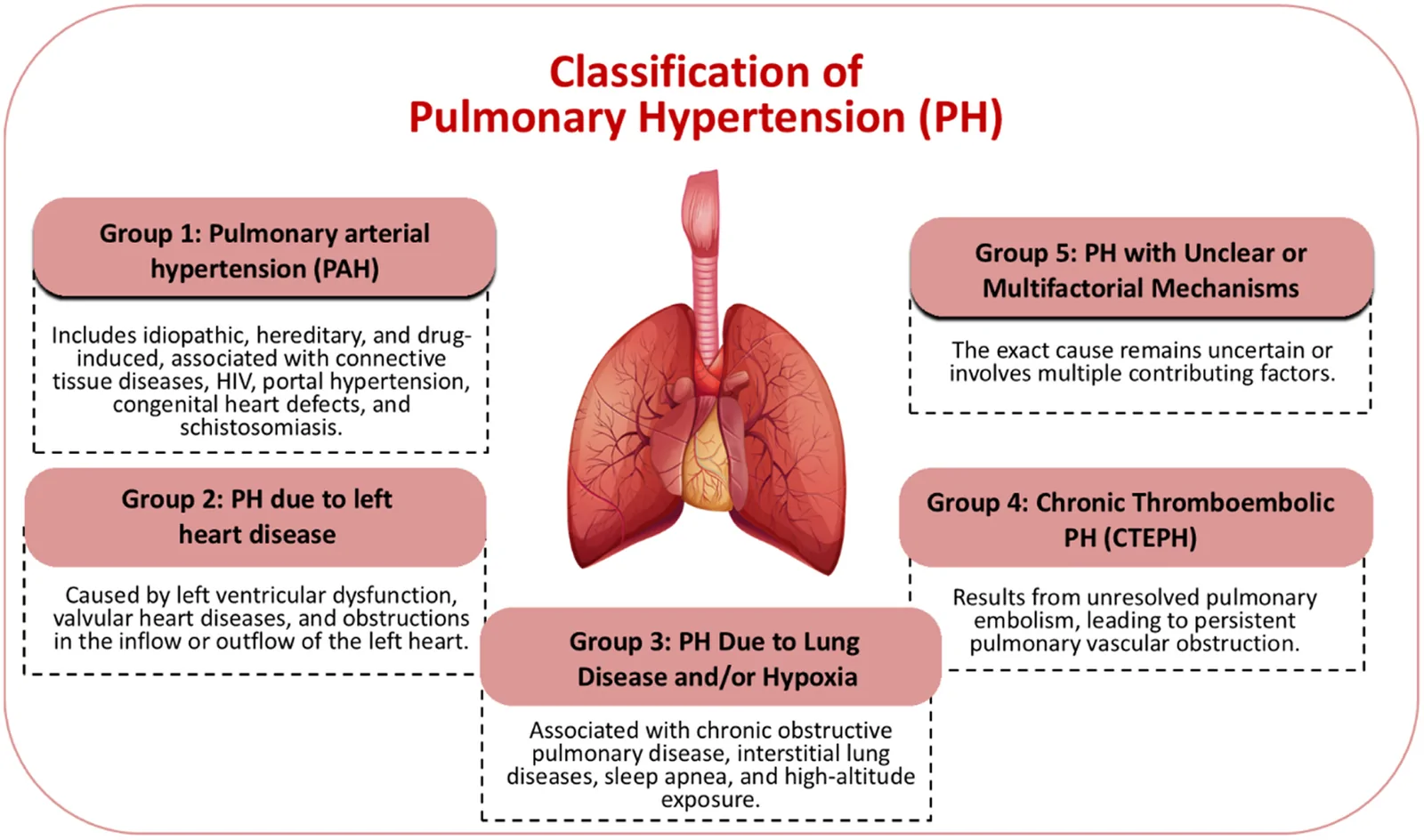
Classification of pulmonary hypertension.

Pulmonary Hypertension (PH) is a progressive and multifactorial condition that can complicate a wide range of cardiovascular and respiratory diseases.^1^ It is characterized by an increased mean Pulmonary Artery Pressure (mPAP) > 20 mmHg at rest.^2^^,^^3^ Epidemiological data suggest that PH affects over 1% of the global population, rising to nearly 10% in individuals over 65-years.^4^ This condition is often associated with various cardiovascular and respiratory effects, manifesting as shortness of breath, decreased exercise tolerance, and heart failure.^5^ Over time, the disease progresses, leading to Right Ventricular (RV) dysfunction and remodeling, which significantly contributes to its poor prognosis.^3^^,^^6^ PH is classified into five distinct groups based on underlying mechanisms, clinical presentation, and management^3^ (Fig. 1). This classification aids in understanding the diverse origins of PH and tailoring appropriate therapeutic strategies.^7^^,^^8^ Despite their heterogeneity, all PH subtypes exert chronic pressure overload on the RV, triggering a sequence of adaptive and maladaptive remodeling.^7^ Initially, the RV hypertrophies to maintain cardiac output, but prolonged afterload leads to dilation, fibrosis, and systolic dysfunction.^9^ This maladaptive response of the RV plays a major role in morbidity and mortality in PH, highlighting the importance of accurate RV assessment.^8^

Reliable evaluation of RV function is essential for determining disease severity and guiding therapeutic decision-making. However, the complex geometry of the RV, its sensitivity to loading conditions, and its interplay with pulmonary circulation make that evaluation challenging.^10^ The emergence of advanced imaging techniques has significantly improved the assessment of RV and pulmonary circulation.^10^, ^11^, ^12^, ^13^, ^14^ While echocardiography and cardiac Magnetic Resonance Imaging (MRI) are routinely used to assess RV structure and function, they are limited in their ability to detect early molecular changes.

In this context, Positron Emission Tomography (PET) has emerged as a valuable non-invasive modality, enabling the assessment of myocardial metabolism, perfusion, inflammation, and innervation. These domains provide complementary insights into the adaptive and maladaptive processes of the RV in PH.^10^

Given the diversity of tracers and methodological approaches, synthesizing how PET has been applied to PH is particularly relevant. To date, no integrative synthesis has comprehensively analyzed these methodological approaches and their clinical relevance. Addressing this gap is critical for improving standardization, interpretation, and translational applicability of PET in PH.

Hence, this integrative review addresses the following questions: i) Which PET tracers are most used for evaluating RV function in PH? ii) How is RV dysfunction evaluated in PET studies? iii) Which quantification methods and metrics are used? iv) How do these parameters correlate with other physiological measures in PH? v) What is the diagnostic and predictive value of PET imaging for PH?

## Materials and methods

The purpose of an integrative review is to provide a synthesis of knowledge and the applicability of results of significant studies to practice.^15^

### Eligibility criteria

This integrative review followed the recommendations of the Preferred Reporting Items for Systematic Reviews and Meta-analyses Extension for Scoping Reviews (PRISMA-ScR) published in 2018.^16^ To ensure methodological rigor and systematically evaluate potential biases, a formal quality appraisal of the included studies was incorporated. Studies were eligible if they included individuals with PH as the primary disease of interest and used PET to assess RV. Studies investigating other primary diseases or using imaging modalities other than PET, including Single-Photon Emission Computed Tomography (SPECT), were excluded. Only articles published in peer-reviewed journals and in English were considered.

### Search strategy

The words and index terms were established using the articles searched before the start of the review. After the aim was decided upon, index terms were established. The terms adjusted for each database are listed in Supplementary Table S1. We used these terms across PubMed, EMBASE, Scopus, and Web of Science databases. Next, the reference lists of all articles included were screened to identify additional relevant studies. Finally, articles published up to July 1, 2024, were considered.

### Study selection

The search records from the four databases were uploaded to Rayyan software,^17^ where duplicates were identified and removed. Two independent reviewers (SP, GSS) screened titles and abstracts for assessment against the inclusion and exclusion criteria. Both reviewers thoroughly assessed the full text against the inclusion criteria. Reviewers resolved disagreements by consensus or through the intervention of a third reviewer (AMMS).

### Data extraction

The reviewers extracted data from each selected article into a dedicated form (SP, GSS). For each article, the following information was extracted: author names, year of publication, complete reference, study design characteristics (e.g., experimental groups, group size), PET protocol (tracer, activity), quantification protocol (software, tracer kinetic model, regions evaluated, segmentation and parameters), main results and limitations.

### Quality and risk of bias assessment

The methodological quality and risk of bias of the included studies were evaluated using the Newcastle-Ottawa Scale (NOS) adapted for observational and cross-sectional studies. Two independent reviewers (SP, GSS) assessed the studies across three key domains: selection of study groups, comparability of the groups, and ascertainment of the exposure or outcome of interest. Studies could be awarded up to nine stars. Discrepancies between reviewers were resolved through discussion and, when necessary, consultation with a third senior reviewer (AMMS). Given that the primary objective of this integrative review was to synthesize and map the breadth of available evidence, studies were not excluded based on their quality score. Instead, the identified methodological limitations were considered in the interpretation of the findings.

## Results

### Literature search

**Fig. 2:**
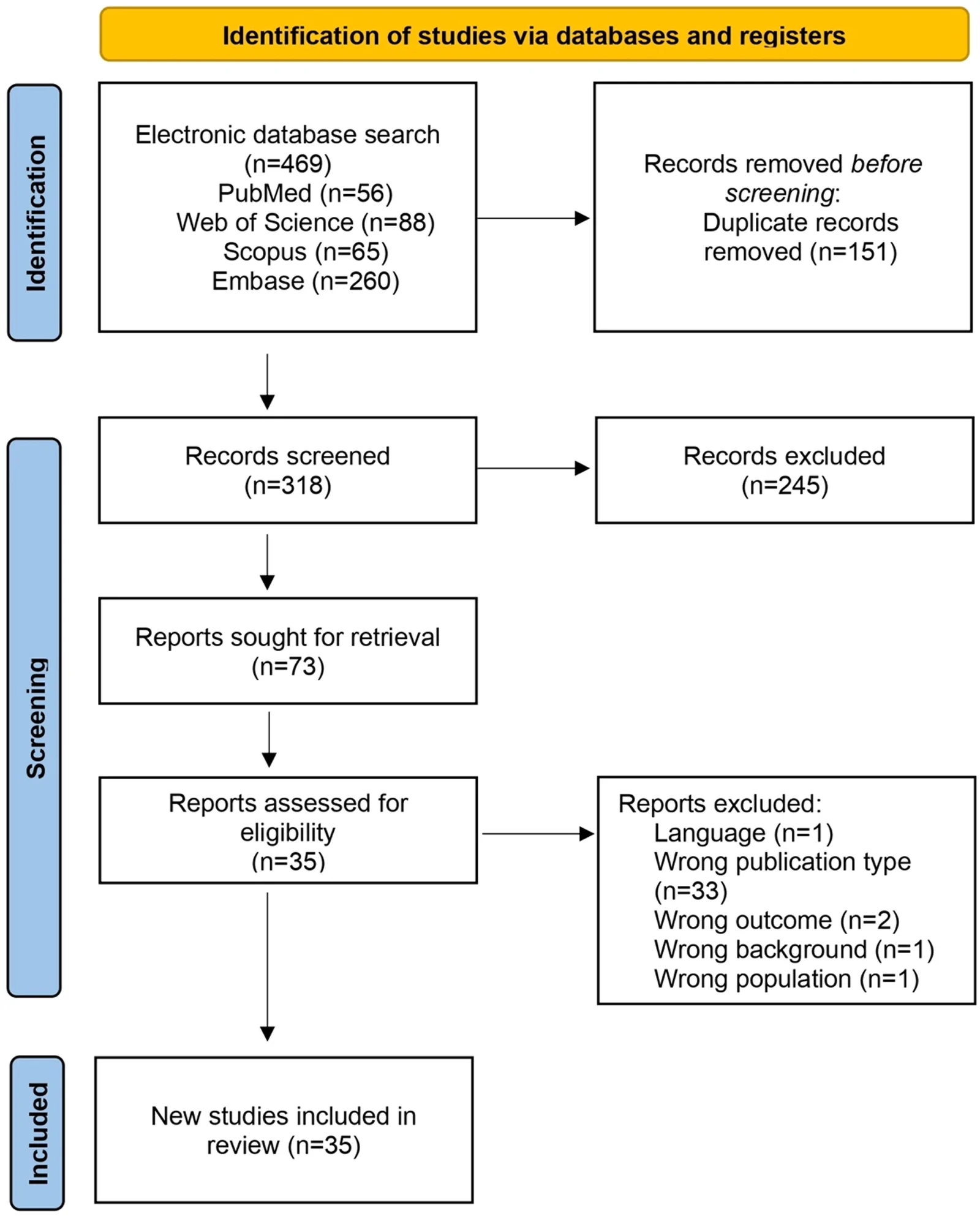
PRISMA flowchart to outline the screening process.

The literature search across various databases yielded 469 publications. This number was reduced to 318 publications after removing duplicate studies, which resulted from the use of multiple databases. Screening of the abstracts resulted in 73 publications that fulfilled all eligibility criteria. The subsequent full-text review excluded an additional 38 papers, resulting in 35 articles that could be used for data extraction and analysis (Fig. 2).

### General description

The 35 included studies spanned a broad timeline: two were published before 2010, ^18^^,^^19^ 13 between 2010 and 2014,^7^^,^^20^, ^21^, ^22^, ^23^, ^24^, ^25^, ^26^, ^27^, ^28^, ^29^, ^30^, ^31^ 10 between 2015 and 2019,^32^, ^33^, ^34^, ^35^, ^36^, ^37^, ^38^, ^39^, ^40^, ^41^ and 10 between 2020 and 2024.^42^, ^43^, ^44^, ^45^, ^46^, ^47^, ^48^, ^49^, ^50^, ^51^

These studies varied in design, with 8 using different patient groups and controls,^21^^,^^22^^,^^25^^,^^41^^,^^42^^,^^47^^,^^48^^,^^50^ while the remaining 27 studies focused on comparing different patient groups based on the PH classification.^7^^,^^18^, ^19^, ^20^^,^^23^^,^^24^^,^^26^, ^27^, ^28^, ^29^, ^30^, ^31^, ^32^, ^33^, ^34^, ^35^, ^36^, ^37^, ^38^, ^39^, ^40^^,^^43^^,^^44^^,^^46^^,^^49^^,^^51^ Different parameters were extracted to quantify the PET image for each type of study. The results are summarized in Supplementary Table S2.

### Quality of included studies

Based on the NOS assessment, the overall methodological quality of the 35 included studies was moderate, with a mean score of 6.1 out of 9 possible stars. Eleven studies were rated as high or moderate-to-high quality (7 to 8 stars), fourteen as moderate quality (6 stars), and ten as moderate-to-low quality (5 stars). A detailed summary of the quality assessment is presented in Supplementary Table S3.

The most common methodological limitation across was the absence of a well-matched healthy control group which frequently reduced the overall NOS score. In several studies, this omission was justified by the inherent technical challenges of quantifying baseline metabolic activity of a normal RV. Additional sources of potential bias included small sample sizes, particularly in studies involving stress protocols or novel PET tracers, and heterogeneity in baseline Pulmonary Arterial Hypertension (PAH) therapies in single-arm longitudinal designs.

### PET tracers and pathophysiological domains

The results of PET tracers used for RV quantification in PH are summarized in Table 1. Multiple tracers are used for PET imaging, each providing insight into distinct pathophysiological aspects of RV adaptation and dysfunction.

**Table 1:** Tracers used for RV quantification for the PH population.

Tracer	N	Study
[^18^F]FDG	27	Hagan et al.,^21^ Sumer et al.,^42^ Fang et al.,^23^ Barton et al.,^44^ Oguz et al.,^33^ Kluge et al.,^18^ Han et al.,^45^ Ahmadi et al.,^34^ Tatebe et al.,^7^ Wang et al.,^24^ Lundgrin et al.,^25^ Oikawa et al.,^19^ Can et al.,^26^ Saygin et al.,^41^ Farha et al.,^47^ Kazimierczyk et al.^48^ Kazimierczyk et al.,^50^ Kazimierczyk et al.,^49^ Kazimierczyk et al.,^51^ Bokhari et al.,^27^ Wang et al.,^35^ Wang et al.,^36^ Wong et al.,^29^ Li et al.,^40^ Yang et al.,^31^ Frille et al.,^39^ Sakao et al.^37^
[^15^O]H_2_O	3	Wong et al.,^20^ Wong et al.,^29^ Wong et al.^30^
[^15^O]O_2_	3	Wong et al.,^20^ Wong et al.,^29^ Wong et al.^30^
[^15^O]CO	3	Wong et al.,^20^ Wong et al.,^29^ Wong et al.^30^
^82^Rb	1	Ohira et al.^38^
[^18^F]FTHA	1	Ohira et al.^38^
[^123^I]-BMIPP	1	Sakao et al.^37^
[^68^Ga]FAPI-04	2	Gu et al.,^43^ Chen et al.^46^
[^13^N]NH_3_	4	Ahmadi et al.,^34^ Bokhari et al.,^27^ Gu et al.,^43^ Ohira et al.^38^
[^11^C]acetate	3	Wong et al.,^20^ Yoshinaga et al. 2014,^22^ Han et al.^45^
[^11^C]HED	1	Rijnierse et al.^32^

[¹⁸F]fluorodeoxyglucose ([¹⁸F]FDG) indicates changes in glucose metabolism and heightened glycolytic activity.^52^ [¹⁸F]FDG is the most frequently used tracer to evaluate RV glycolytic metabolism, appearing in 27 studies. The selection is related to the fact that, in PH, chronic pressure overload causes a metabolic shift in the RV from oxidative phosphorylation to glycolysis.^52^ Initially, this change helps maintain ATP production under stress, but long-term dependence on glycolysis leads to maladaptive remodeling, hypertrophy, and eventual systolic dysfunction.^53^

Tracers such as [¹³N]ammonia ([¹³N]NH₃), [¹⁵O]H₂O, and ^82^Rb directly assess Myocardial Blood Flow (MBF), also enabling the estimation of Myocardial Oxygen consumption (MVO₂), providing insight into perfusion-oxygen mismatch.^27^^,^^54^^,^^55^ [¹¹C]acetate, evaluated in three studies, provides complementary information on oxidative metabolism and myocardial efficiency. PET with [^11^C]acetate enables the indirect quantification of MVO₂ through clearance kinetics, which correlates with RV workload and efficiency.^20^ Perfusion tracers ([¹³N]NH₃, [¹⁵O]H₂O, ^82^Rb), employed in eight studies, reveal heterogeneous RV perfusion patterns likely secondary to microvascular remodeling and regional ischemia.^27^^,^^54^^,^^55^

Fatty acid tracers, including [¹⁸F]FTHA and [¹²³I]BMIPP, used in one study, indicate β-oxidation impairment and reduced lipid utilization in the pressure-overloaded RV.^56^ More recently, [⁶⁸Ga]fibroblast activation protein inhibitor ([⁶⁸Ga]FAPI) has shown promise in detecting fibroblast activation, reflecting fibrotic remodeling processes in advanced disease stages.^43^ Fibrosis imaging with [⁶⁸Ga]FAPI (two studies) detected early fibroblast activation and interstitial fibrosis, which contribute to RV stiffening and functional impairment before structural changes are detectable with conventional imaging.^43^

Finally, sympathetic innervation tracers ([¹¹C]HED), which reveal regional dysregulation of autonomic input and potential mechanistic links to arrhythmogenesis and contractile dysfunction, are investigated in a small number of studies.^57^ Tracers assessing sympathetic innervation, such as [¹⁸F]LMI1195, have been used in PH and cardiac sarcoidosis, linking autonomic dysfunction to RV impairment.^57^ The inclusion of innervation tracers is particularly relevant because neurohormonal dysregulation represents an early and often underexplored mechanism of RV dysfunction.

To facilitate comparison between these tracers, Table 2 summarizes their principal molecular targets, physical half-lives, clinical advantages, and practical limitations. While [^18^F]FDG remains the most accessible tracer for detecting the glycolytic shift associated with RV hypertrophy, its limited molecular specificity and the need for strict metabolic preparation protocols remain important challenges. In contrast, tracers such as [^11^C]acetate, [^15^O]H₂O, and [^11^C]HED allow highly quantitative assessments of oxidative metabolism, myocardial perfusion, and sympathetic innervation, respectively, but their broader clinical use is constrained by short physical half-lives and. Emerging targeted tracers, such as[^68^Ga]FAPI provide the opportunity to direct visualize fibroblast activation and early of fibrotic remodeling.

**Table 2:** Comparison of PET tracers used in right ventricular assessment for pulmonary hypertension.

PET Tracer	Target / Mechanism	Physical Half-life	Clinical & Biophysical Advantages	Practical Limitations & Disadvantages
[^18^F]FDG	Glucose metabolism (Glycolysis)	109.8 min	Widely available; excellently suited for detecting the glycolytic shift in the hypertrophied RV.	Lacks specificity to differentiate metabolism from severe inflammation; requires strict and complex dietary preparation (e.g., prolonged fasting or glucose loading) to suppress physiological uptake.^25^, ^31^
[^11^C]acetate	Oxidative metabolism	20.4 min	Allows exact quantification of myocardial oxygen consumption and mechanical efficiency.	Requires an on-site cyclotron due to its short half-life; limited availability; highly complex kinetic modeling subject to partial volume effects.^20^, ^22^
[^15^O]H_2_O	Myocardial blood flow (Perfusion)	2.0 min	Gold standard for absolute perfusion quantification; freely diffusible without metabolic trapping.	Extremely short half-life mandates an on-site cyclotron directly linked to the PET suite; requires highly complex dynamic acquisition protocols.^20^
[^68^Ga]FAPI	Fibroblast activation (Fibrosis)	67.7 min	Direct molecular imaging of active fibrotic remodeling; potentially detects changes earlier than CMR delayed enhancement.	Limited availability (requires specific generators) and high cost; incremental prognostic value over CMR in pH is not yet fully validated in large cohorts. 43, 46
[^11^C]HED	Sympathetic innervation	20.4 min	Quantifies autonomic dysfunction and neurohormonal overactivation in the failing RV.	Requires an on-site cyclotron; utilization is mostly restricted to highly specialized research centers; high procedural cost.^32^
[¹³N]NH₃	Myocardial Blood Flow (MBF) / Perfusion	10 min	Feasible modality for quantifying right ventricular blood flow; provides high-resolution images to assess RV perfusion-oxygen mismatch.	Requires an on-site cyclotron; requires dynamic imaging acquisition and the application of a 2-compartment kinetic model.^27^, ^34^
^82^Rb	Myocardial Blood Flow (MBF) / Perfusion.	76s	Generator-produced tracer, which overcomes the need for an on-site cyclotron and allows blood flow quantification to be more widely available.	Similar to other perfusion tracers, it relies on dynamic imaging and automated software for complex kinetic modeling to quantify absolute blood flow.^27^

### PH regions of interest

Various distinct regions, including the RV, LV, and lungs, are examined in cardiac diseases. Usually, the LV is the region or volume of interest (ROI or VOI) most frequently evaluated in cardiac pathologies. In PH, the lungs are also affected due to pulmonary vascular inflammation and remodeling, which has led to some studies to define ROIs in this area for assessment. However, since this review focuses on RV quantification, only RV and LV assessments will be included, as some studies calculate the uptake ratio between the RV and LV.

The delimitation of ROIs for analyzing the RV and LV varies across studies, with many not specifying the methods used. Most studies position the ROIs on the free walls of the RV and LV, while some also include the interventricular septum when defining these areas. Typically, between five and 14 circular ROIs are used in transaxial sections to obtain the tracer uptake metric.^21^ In some cases, fixed shapes, such as circular or square ROIs, are used, positioned at the center of the RV or LV.^24^

A common approach involves manually drawing ROIs in at least three coronal planes according to the longest cardiac axis (apical, medial, and basal).^32^ Many studies employ Computed Tomography (CT) scans as a guide to define RV regions, leveraging detailed imaging to assist in accurately delineating the areas of interest.^58^, ^59^, ^60^ Other studies combine MRI with PET, allowing for even more precise ROI delineation.^48^ The combination of imaging techniques has become increasingly valued for enhancing the accuracy and reliability of measurements. Chen et al.^46^ fused PET and CT images without iodine contrast-enhancement to manually define the ROI around the RV wall, from which the maximum SUV was derived. If no visible uptake was detected, a 5 mm circular ROI was placed on the free wall of the RV at the aortic root level to obtain the maximum SUV.

One alternative approach to delineate cardiac ROIs uses areas with more than 60% of maximum uptake.^34^ Depending on the PET tracer and acquisition method, the early frames are initially merged to delineate the ROIs, which are then applied to the dynamic scan to extract the time-activity curves for kinetic analysis.^20^

### PET quantitative parameters

PET image quantification relies predominantly on semiquantitative analysis using SUV metrics and parameters calculated from kinetic modeling. SUV analysis is the most used method, particularly for studies utilizing [^18^F]FDG PET. SUV normalizes tracer uptake based on injected activity and patient characteristics. Most studies reported the mean (SUV_mean_) and maximum SUV (SUV_max_), with some including the RV/LV SUV ratio (SUV_RV/LV_) to improve inter-patient comparability. The SUV_max_, representing the highest voxel SUV within an ROI, is commonly used in clinical settings due to its simplicity. Still, it is sensitive to noise and may overestimate uptake in small abnormal regions.^61^ Conversely, SUV_mean_, the average SUV within a defined ROI, provides a more stable metric that mitigates the influence of localized high-intensity voxels.^62^

Beyond the SUV, additional semiquantitative parameters are applied to capture more specific physiological aspects. The Tissue-to-Blood Ratio (TBR) and Retention Index (RI) are applied in some studies. TBR normalizes RV tracer uptake to background blood pool activity and is used in studies employing [^18^F]FDG and [^68^Ga]FAPI PET to enhance signal-to-noise contrast. RI, derived from [^11^C]HED PET, is used to evaluate cardiac sympathetic innervation, although its clinical relevance in PH remains under investigation.^32^

Kinetic modeling (KM) offers a more comprehensive and biologically relevant understanding of right ventricular physiology through the analysis of tracer dynamics over time. For instance, Patlak linearization model and the irreversible two-tissue compartment model (2T3k) are commonly used to quantify Myocardial Glucose Uptake (MGU) using [^18^F]FDG. This approach allows calculation of metabolic rates and provide insight into energy utilization by the RV under chronic pressure overload.^27^ Similarly, [¹¹C]acetate PET employs monoexponential clearance (K_mono_) to estimate MVO₂, reflecting the efficiency of oxidative metabolism and revealing how energetic supply matches functional demand.^20^ MBF quantification during rest and stress protocols is assessed with [^13^N]NH_3_ PET and [^15^O]H_2_O PET using one-tissue compartment models (1T2k), enabling identification of perfusion deficits that may precede detectable contractile dysfunction.^27^^,^^30^ These KM methods approach, while offering more physiologically meaningful assessments, require dynamic imaging acquisition, complex analysis, and careful definition of ROIs, which limits their widespread adoption in clinical practice. Table 3 summarises the quantification methods and outcome parameters used in the studies related to RV dysfunction.

**Table 3:** Quantification methods and outcome parameters found in the reviewed articles.

Study	Tracer	Method	PET metrics	Kinetic model	Outcome PET parameters
Hagan et al.,^21^ Lundgrin et al.^25^	[^18^F]FDG	Semiquantitative	TBR	‒	TBR
Oikawa et al.,^19^ Tatebe et al.,^7^ Frille et al.,^39^ Farha et al.,^47^ Lundgrin et al.,^25^ Han et al.^45^	[^18^F]FDG	Semiquantitative	SUV	‒	SUV_mean_
Fang et al.,^23^ Wan et al.,^24^ Yang et al.,^31^ Sakao et al.,^37^ Saygin et al.,^41^ Ahmadi et al.,^34^ Oguz et al.,^33^ Kazimierczyk et al.,^48^ Kazimierczyk et al.,^50^ Kazimierczyk et al.,^49^ Kazimierczyk et al.^51^	[^18^F]FDG	Semiquantitative	SUV_mean_	‒	SUV_RV/LV_
Can et al.^26^	[^18^F]FDG	Semiquantitative	SUV_max_	‒	SUV_RV/LV_
Li et al.^40^	[^18^F]FDG	Semiquantitative	cRVSUVg	‒	SUV_RV/LV_
Bokhari et al.^27^ Wang et al.^35^ Wang et al.^36^	[^18^F]FDG	Quantitative	‒	Patlak	MGU, MGUR
Barton et al.^44^	[^18^F]FDG	Quantitative	‒	2T3k	K_i_
Kluge et al.^18^	[^18^F]FDG	Quantitative	‒	Patlak	MGUR
Han et al.^45^	[^11^C]acetate	Quantitative	‒	K_mono_, 1T2k	K_1_, MVO_2_
Wong et al. ,^20^ Yoshinaga et al.^22^	[^11^C]acetate	Quantitative	‒	K_mono_	MVO_2_
Wong et al. ,^28^ Wong et al. ,^29^ Wong et al.^20^	[^15^O]H_2_O, [^15^O]O_2_, and [^15^O]CO	Quantitative	‒	1T2k	MBF
Gu et al.^43^	[^68^Ga]FAPI	Semiquantitative	SUV	‒	SUV_max_
Chen et al.^46^	[^68^Ga]FAPI	Semiquantitative	SUV_max_	‒	TBR RV
Rijnierse et al.^32^	[^11^C]HED	Semiquantitative	TAC, AUC	‒	RI
Bokhari et al.^27^	[^13^N]NH_3_	Quantitative	‒	2T3k	MBF
Ohira et al.^38^	^82^Rb or [^13^N]NH_3_	Semiquantitative	SUV_mean_	‒	SUV_mean_, SUV_RV/LV_

AUC, Area Under the Curve; cRV SUVg, Corrected Right Ventricle SUV glucose-loading; KM, Kinetic modeling; MGU, Myocardium Glucose Uptake; MGUR, MGU (RV/LV) Ratio; MVO_2_, Myocardial Oxygen consumption; RI, Retention Index; SUV, Standardized Uptake Value; SUV_RV/LV_, Standardized Uptake Value ratio; TAC, Time-Activity Curves; TBR, Tissue-to-Blood Ratio.

### PET and physiological parameters

Several studies examine the correlation between PET-derived parameters and clinical or hemodynamic measures of PH severity. These correlations encompass hemodynamic parameters, such as mPAP, systolic Pulmonary Arterial Pressure (sPAP), and Pulmonary Vascular Resistance (PVR), all critical indicators of right ventricular overload. Functional parameters, such as Right Ventricular Ejection Fraction (RVEF) and Tricuspid Annular Plane Systolic Excursion (TAPSE), as well as biomarkers, such as the 6-Minute Walk Distance (6MWD) and Right Ventricular Systolic Work Index (RVSWI), are also correlated with PET-derived metrics.

Table 4 summarizes the significant correlations (*r* ≥ 0.7 and p < 0.05) between PET metrics and key physiological parameters. Correlations above 0.7 indicate a high correlation, while values exceeding 0.9 represent a very high correlation.^63^ Supplementary Tables S4 and S5 provide moderate correlations (values between 0.5 and 0.7) and low or negligible correlations (*r* < 0.5).

**Table 4:** High correlations (*r* ≥ 0.7 and p < 0.05) between PET metrics and clinical measures of PH severity from the reviewed articles.

Study	Tracer	Group	Sample size (n)	PET metrics	Cardiovascular parameters	Correlation (*r*)
Wong et al.^28^	[^15^O]H_2_O, [^15^O]O_2_, [^15^O]CO	PH due to left heart disease and CTEPH	n = 18	MVO_2_	sPAP	0.73
					RPP	0.74
Wong et al.^29^	[^15^O]H_2_O, [^15^O]O_2_, [^15^O]CO, and [^18^F]FDG	PH due to left heart disease and CTEPH	n = 16	RV efficiency (%)^a^	RV ejection fraction %	−0.81
					PS-IVP	0.76
Sumer et al.^42^	[^18^F]FDG	IPAH, and CTEPH	Patients: n = 17; Controls: n = 30	RV SUV	mPAP	0.70
Kluge et al.^18^	[^18^F]FDG	Primary PH; CTEPH; PH of other cause	n = 30	SUVR_mean_	PAR	0.77
Ahmadi et al.^34^	[^18^F]FDG	CTEPH	n = 6	SUVR	RVEF	−0.82
					mPAP	0.94
Lundgrin et al.^25^	[^18^F]FDG	PAH	Patients: n = 14; Controls: n = 6	TBR	Systolic ecocentricity index	0.76
					RV fractional area change	−0.85
					RV outflow tract velocity time integral	−0.75
Chen et al.^46^	[^68^Ga]FAPI-04	CTEPH	n = 13	TBR	RV wall	−0.80
Oikawa et al.^19^	[^18^F]FDG	PH	n = 24	SUV	mPAP	0.78
					RV wall stress	0.74
Oikawa et al.^19^	[^18^F]FDG	PH	n = 24	% change of SUV (after-before intervention)	% change in the PVR	0.78
					RV systolic wall stress	0.76
Can et al.^26^	[^18^F]FDG	PAH	Patients: n = 23; Controls: n = 16	SUVR	PAPs	0.87
					TAPSE	−0.80
					6MWD	−0.74
Saygin et al.^41^	[^18^F]FDG	PH	Patients: n = 30; Controls: n = 8	SUVR	RVES	0.70
					RV GPSS	0.70
Kazimierczyk et al.^50^	[^18^F]FDG	PAH	Patients: n = 26; Controls: n = 12	SUVR	mPAP	0.77
					RVSWI	0.72
Kazimierczyk et al.^49^	[^18^F]FDG	PAH	n = 18	SUVR	RVEF	0.78
Kazimierczyk et al.^51^	[^18^F]FDG	PAH	n = 20	SUVR	mPAP	0.79
					PVR	0.82
					PAC	−0.73
					RVSWI	0.77
Bokhari et al.^27^	[^18^F]FDG	IPAH	n = 16	MGUR	PA systolic	0.75
					mPAP	0.87
Ohira et al.^38^	[^18^F]FDG	PAH	n = 17	SUVR	mPAP	0.71

6MWD, 6-Minute Walk Distance; CTEPH, Chronic Thromboembolic Pulmonary Hypertension; IPAH, Idiopathic Pulmonary Arterial Hypertension; mPAP, Mean Pulmonary Artery Pressure; MGUR, Myocardial Glucose Uptake Ratio (RV/LV); MVO2, Myocardial Oxygen Consumption; PA systolic, Pulmonary Artery systolic; PAC, Pulmonary Arterial Compliance; PAH, Pulmonary Arterial Hypertension; PAPs, Pulmonary Artery Pressures; PAR, Pulmonary Right ventricular; PH, Pulmonary Hypertension; PS-IVP, Postsystolic Isovolumic Period; PVR, Pulmonary Vascular Resistance; RPP, Rate-Pressure Product; RV ED, Right Ventricular End-Diastolic; RV ES, Right Ventricular End-Systolic; RV GPSS, Right Ventricular Global Peak Systolic Strain; RVEF, Right Ventricular Ejection Fraction; RVSWI, Right Ventricular Systolic Work Index; SUV, Standardized Uptake Value; SUVR, Standardized Uptake Value Ratio; TAPSE, Tricuspid Annular Plane Systolic Excursion; TBR, Tissue-to-Blood Ratio.

a RV efficiency (%) is calculated from the ratio of RV power output from hemodynamic data and MVO_2_ from PET.^29^

### PET metrics as a biomarker

Recent studies have reported associations between PET-derived metrics and indicators of disease severity in PH. Parameters such as SUV and SUVRV/LV may provide insights into metabolic alterations in the RV and their relationship with clinical status. Table 5 summarizes the studies that evaluated PET metrics as potential prognostic markers in PH, including the reported cut-off values. However, the available evidence is largely derived from small, single-center observational studies, and the reported relationships should therefore be interpreted with caution.

**Table 5:** Summary of how PET metrics help with clinical PAH outcomes.

Study	Tracer	Comparative group	Sample size (n)	PET Metrics	Cut-off value	Findings	Hazard Ratio (95% CI)
Tatebe et al.^7^	[^18^F]FDG	PAH vs. CTEPH	n = 27	RV SUV	≥ 8.3	Associated with worse prognosis and higher all-cause mortality	1.25 (1.04 - 1.51), p = 0.017
Kazimierczyk et al.^50^	[^18^F]FDG	PAH (class I) vs. control group	Patients: n = 26; Controls: n = 12	SUV_RV/LV_	> 1.0	Associated with a worse prognosis and a lower survival rate	‒
Kazimierczyk et al.^48^	[^18^F]FDG	PAH (class II and III) vs. control group	Patients: n = 27; Controls: n = 12	SUV_RV/LV_	1.0	SUVR was significantly higher in PAH patients compared to healthy controls and correlated with RV dysfunction parameters	17.84 (1.48– 214.8), p = 0.02
Kazimierczyk et al.^51^	[^18^F]FDG	PAH (class I) vs. control group	n = 20	SUV_RV/LV_	> 0.54	Associated with a worse prognosis in PAH	Log-rank; p = 0.0007
Farha et al.^47^	[^18^F]FDG	IPAH vs. control group	Patients: n = 30; Controls: n = 12	SUV_RV/LV_	> 0.8	Associated with a more severe disease phenotype, higher RV SP, RA pressure, endothelin-1 levels, and worse RV function	‒
Li et al.^40^	[^18^F]FDG	IPAH	n = 45	SUV_RV/LVf_	1.44	Associated with a worse prognosis in IPAH patients	SUV_R__V/LV__f_ HR = 1.027, p = 0.043
				SUV_RV/LVg_	1.20		SUV_R__V/LV__g_ HR = 1.020; p = 0.035
Gu et al.^43^	[^68^Ga]FAPI	PAH	n = 16	SUV_RV/LV_	> 1.2	Associated with greater disease severity and worse outcomes in PAH	2.3 (1.1–4.5); p = 0.03
Fang et al.^23^	[^18^F]FDG	IPAH vs. CHD	n = 67	SUV_RV/LV_	> 1.2	Correlates with significantly lower survival rates in IPAH	2.4; p < 0.05

CHD, Congenital Heart Disease; CI, Confidence Interval; CTEPH, Chronic Thromboembolic Pulmonary Hypertension; IPAH, Idiopathic Pulmonary Arterial Hypertension; PAH, Pulmonary Arterial Hypertension; RV SP, Right Ventricle Systolic Pressure; SUV, Standardized Uptake Value; SUV_RV/LV_, Standardized Uptake Value Ratio; SUV_RV/LVf_, Standardized Uptake Value Ratio Fasting; SUV_RV/LVg_, Standardized Uptake Value Ratio glucose-loading.

## Discussion

PET imaging has emerged as a valuable tool for assessing RV function in PH, offering unique insights into myocardial metabolism, perfusion, fibroblast activation, and fibrotic remodeling. These advanced molecular imaging techniques may enhance the understanding of PH pathophysiology and disease progression. However, heterogeneity in tracers and imaging protocols across studies introduces significant variability, impairs data interpretation, and limits inter-study comparability. This section discusses key challenges in PET imaging of PH, focusing on PET tracers, quantification metrics, the prognostic significance of PET metrics, and implications for clinical practice and research.

### PET tracers

Among the most frequently used PET tracers employed to investigate distinct aspects of RV pathophysiology in PH are [^18^F]FDG, [^11^C]acetate, [^15^O]H_2_O, and [^68^Ga]FAPI. [^18^F]FDG, a glucose metabolism tracer, is the most extensively investigated tracer in RV assessment. Chronic pressure overload in PH induces a metabolic shift in the RV from oxidative phosphorylation toward glycolysis, reflecting an adaptive response to maintain ATP production under increased workload.^53^^,^^56^ Elevated RV [^18^F]FDG uptake correlates with hemodynamic severity, including mPAP and PVR, and with indices of RV dysfunction, such as reduced RVEF.^21^^,^^42^ However, interpretation of [^18^F]FDG uptake is influenced by patient preparation (fasting, heparin administration, or dietary interventions), physiological myocardial uptake, and partial volume effects due to the thin RV wall. These factors may explain discrepancies across studies in the magnitude of tracer accumulation and its correlation with clinical parameters.

Conversely, [^11^C]acetate indirectly measures the MVO_2_, with the clearance rate reflecting oxidative metabolism. This is particularly relevant in idiopathic PAH, where altered energy metabolism is a hallmark.^20^^,^^22^ Compared to [^18^F]FDG, [^11^C]acetate offers a complementary perspective, capturing oxidative rather than glycolytic metabolism. However, dynamic acquisitions and kinetic modeling requirements limit its routine clinical use and may contribute to variability in inter-study comparisons.

Perfusion tracers, including ^82^Rb, [¹³N]NH₃, and [¹⁵O]H₂O, are used to assess MBF, capturing potential ischemia contributing to RV dysfunction, revealing areas susceptible to ischemia due to increased wall stress and microvascular remodeling. These measurements provide mechanistic insight into energy supply-demand mismatch in the pressure-overloaded RV, which may underlie contractile dysfunction.^27^^,^^34^ More recently, [^68^Ga]FAPI, a biomarker of fibroblast activation, enables the detection of myocardial fibrosis. Fibroblast activity, a hallmark of pathological remodeling, leads to excessive extracellular matrix deposition and contributes to RV stiffening and functional impairment.^64^ Increased [^68^Ga]FAPI uptake in the RV correlates with disease severity and may offer a prognostic value for patients with advanced PH by identifying fibrotic changes that are not easily detected with anatomical imaging.^43^

### Quantification of RV dysfunction

PET-derived metrics, such as SUV_mean_, SUV_max_, SUV_RV/LV_, and TBR, have been extensively used to quantify RV metabolic and structural alterations.

Elevated [^18^F]FDG uptake in the RV has a strong correlation with hemodynamic parameters, including mPAP, PVR, and RV systolic indices. SUV measurement shows a strong correlation with mPAP (*r* = 0.70 to 0.78),^19^^,^^42^ reflecting increased metabolic activity in response to pressure overload. [^18^F]FDG SUV_RV/LV_ correlates with sPAP (*r* = 0.73) and RV efficiency indices (*r* = −0.81), reinforcing its role as a marker of disease severity and ventricular strain. Similar findings are reported by Ahmadi et al. and Kazimierczyk et al., showing negative correlations between [^18^F]FDG SUV_RV/LV_ and RVEF (*r*=−0.82), and positive correlations with mPAP (*r* = 0.79) and PVR (*r* = 0.82), further emphasizing the role of PET imaging in monitoring RV dysfunction.^34^^,^^51^

[^18^F]FDG TBR metric provides a regional assessment of RV metabolism relative to the LV. Lundgrin et al.^25^ demonstrated that TBR correlates with RV fractional area change (*r* = −0.85) and RV outflow tract velocity time integral (*r* = −0.75). Increases in RV TBR over 12-months are associated with worsening RV function and clinical deterioration, underscoring its potential as a non-invasive biomarker for monitoring disease progression.

Beyond [^18^F]FDG, other tracers offer complementary mechanistic and quantitative insights, but correlations with hemodynamic, functional, and biomarker parameters are often moderate and variable. [¹¹C]acetate-derived K_mono_ showed moderate correlations with PVR (*r* = 0.58) and weaker associations with mPAP and NT-proBNP (*r* = 0.46 and 0.26, respectively) in PH due to left heart disease and CTEPH.^31^ [¹¹C]HED metrics showed moderate correlations with RVEF, wall tension, CO, and PVR (*r* = 0.57–0.69) in idiopathic PAH.^40^ [¹⁵O]-labeled tracers provided stronger correlations for RV efficiency and MVO₂ with sPAP and rate-pressure product (*r* = 0.73–0.76), although derived from small cohorts.^29^ [⁶⁸Ga]FAPI showed moderate correlations with RV fractional area change (*r* = −0.68) and biomarkers (SUV_max_ vs. TPR and NT-proBNP *r* = 0.53–0.61) in CTEPH and PAH populations.^43^^,^^46^ These data indicate that, while these tracers enrich understanding of RV pathophysiology, their quantitative and prognostic utility remains to be validated in larger, more diverse cohorts.

However, the major source of variability across studies is the heterogeneity in the delineation of the region of interest. The selection of anatomical planes for imaging quantification introduces an additional layer of complexity. Some studies quantify the regions exclusively on midventricular transaxial slices.^22^ In contrast, others incorporate apical, medial, and basal slices on the coronal plane to capture a more comprehensive profile of tracer distribution in the heart.^23^ Such differences lead to substantial variations in quantitative results, further complicating cross-study comparisons.^65^ The reproducibility of the size and placement of the ROI influences SUV_mean_ and SUV_peak_, requiring proper standardization or automation. Consequently, the choice of SUV parameter can fundamentally change the assessment of disease progression or response to treatment in most cases.^66^

Furthermore, two significant technical limitations persist in the literature: the omission of Partial Volume Correction (PVC) applied to PET images, and the lack of motion correction in dynamic studies. Of 35 studies analyzed, 74% did not implement PVC methods, and 51% lacked motion correction. Due to the limited spatial resolution of PET systems (∼4–6 mm), the partial volume effect is significant, and signal spill-out causes underestimation of tracer uptake.^67^ It can substantially distort quantitative metrics, such as SUV and k₂.^68^, ^69^, ^70^ Respiratory and cardiac motion introduce misalignment between PET data and attenuation correction maps, degrading spatial resolution.^70^^,^^71^ Studies applying PVC and motion-compensated reconstruction, such as PET-MR with PSF-based PVC, have demonstrated up to 30.9% reduction in wall thickness and 82% increase in myocardium-to-blood ratio.^72^ The routine adoption of PVC and motion correction is critical to enhance reproducibility, accuracy, and clinical applicability of PET-derived RV metrics.

### Prognostic value of PET imaging

Evidence supporting the potential prognostic utility of PET imaging in PH is growing. Elevated RV SUV and SUV_RV/LV_, particularly in [^18^F]FDG and [^68^Ga]FAPI PET images, are associated with increased mortality and adverse outcomes in PH patients.^48^^,^^50^ RV SUV higher than or equal to 8.3 is associated with higher all-cause mortality HR = 1.25; (95% CI 1.04–1.51; p = 0.017),^7^ while SUV_RV/LV_ > 1 is associated with a worse prognosis and lower survival rate HR = 17.84 (95% CI 1.48–214.8; p = 0.02).^48^ Moreover, an SUVR > 0.54 indicates a worse outcome for PAH subjects.^51^

For fibrotic remodeling tracers, elevated [^68^Ga]FAPI uptake (SUV_RV/LV_ > 1.2) is associated with adverse clinical outcomes, including higher mortality HR = 2.3; (95% CI 1.1–4.5; p = 0.03).^23^^,^^43^ Fibrosis, which causes irreversible changes in myocardial structure and function, is a key factor in RV failure and patient survival, further underscoring the clinical value of [^68^Ga]FAPI PET imaging in PH.^70^

### Implications for clinical practice

Clinically, PET enables the non-invasive assessment of RV pathophysiology, providing insights into metabolic and molecular alterations that may precede overt structural remodeling. However, echocardiography and Cardiac Magnetic Resonance (CMR) remain the established modalities for evaluating RV anatomy, volumetry, and global function in PH.^73^ In particular, CMR techniques such as T1 mapping and Extracellular Volume (ECV) quantification offer robust and reproducible markers of diffuse myocardial fibrosis and have demonstrated strong associations with right ventricular hemodynamics and dysfunction in PH.^74^ In this context, PET imaging should currently be considered a complementary modality rather than a replacement for established structural imaging techniques.

Among PET tracers, [^18^F]FDG is the most extensively studied and validated, showing consistent associations with hemodynamic markers of disease severity. Nevertheless, outcome studies directly linking PET-derived metrics to hard clinical endpoints remain limited. While [^15^O]-labelled tracers offer comprehensive multiparametric assessments of myocardial perfusion and oxygen consumption, their clinical implementation is restricted use is limited by logistical barriers, including the need for an on-site cyclotron, the isotope’s ultra-short half-life, and complex acquisition protocols involving multiple injections and potential arterial sampling.^69^ [^11^C]acetate provides a more practical approach to estimating oxidative metabolism through a single dynamic acquisition without arterial cannulation, although correlations with clinical indices remain are low to moderate (Table 4 and Supplementary Table 5). More recently, [^68^Ga]FAPI enables in vivo assessment of fibroblast activation, a key process in myocardial fibrotic remodeling. Increased FAPI uptake has shown moderate to strong associations with hemodynamic parameters and clinical outcomes, suggesting that it may serve as a sensitive marker of advanced RV remodeling.

Despite these promising findings, the incremental prognostic value of PET over established imaging modalities has not yet been clearly demonstrated. Most currently available studies evaluate PET-derived metrics in isolation or alongside basic echocardiographic parameters, rather than through direct head-to-head comparisons with advanced CMR-based fibrosis markers. As a result, it remains uncertain whether the molecular sensitivity offered by PET tracers such as [^68^Ga]FAPI translates into a clinically meaningful advantage over established structural imaging techniques.

Although there are currently no formal guidelines recommending PET in the routine evaluation of PH, available evidence suggests that tracers such as [¹⁸F]FDG and [⁶⁸Ga]FAPI may provide complementary information regarding metabolic stress and fibrotic remodeling. Elevated RV [¹⁸F]FDG SUV and increased SUV_RV/LV_ , as well as higher [^68^Ga]FAPI uptake, have been associated with adverse outcomes in several observational studies. Together, these tracers may offer additional insights into RV maladaptation; however, prospective studies incorporating multiparametric imaging approaches will be necessary to determine their true clinical utility and incremental value in risk stratification.

### Limitations and future perspectives

Collectively, there are methodological differences in PET quantification for PH assessment, ranging from the shape and size of regions of interest to the choice of anatomical planes and kinetic modeling techniques, highlighting the lack of PET imaging standardization. Achieving reproducibility and comparability across studies becomes challenging without a consensus regarding the delineation of regions of interest, thereby affecting the precision of PET imaging quantification and obscuring true physiological or pathological trends.

The interpretation and generalization of our findings are limited by certain aspects of the reviewed studies. Given the predominantly observational and frequently cross-sectional design of the included studies, the interpretation of associations between PET-derived metrics and clinical outcomes should be approached with caution, and causal or prognostic inferences remain limited. One primary limitation is the variability of the PET-based metrics, even when the same tracer is used for evaluation. Moreover, [^18^F]FDG, [^11^C]acetate, and [^68^Ga]FAPI highlight distinct molecular processes, making it challenging to compare and identify common PET metrics. Furthermore, several authors reported that the small sample size limits the generalizability of their findings.

Future efforts should concentrate on establishing consensus-based imaging protocols that include PVC and motion correction as standard practices. To move beyond generic recommendations, actionable steps must include standardizing specific SUV normalization methods (e.g., blood pool referencing) and establishing strict ROI delineation protocols that consistently encompass basal, mid, and apical slices. Head-to-head comparisons of different tracers within the same cohort would clarify their relative effectiveness in identifying PH phenotypes and monitoring disease progression. Similarly, comparisons of different tracers within the same cohort would contribute to the same questions. Such research could enhance the role of PET imaging in diagnosing and tracking PH, thereby providing a more robust foundation for future clinical translation. Moreover, integrating machine learning techniques for ROI automation and parameter prediction may further improve reproducibility and clinical relevance.

## Conclusion

PET imaging is a valuable non-invasive modality for evaluating right ventricular function in pulmonary hypertension. It enables quantification of metabolic, perfusion, fibrotic, and innervation biomarkers that closely reflect the disease severity and prognosis. While elevated SUV and SUV_RV/LV_ have been frequently reported to be associated with adverse outcomes, definitive predictive value remains unproven. Nevertheless, methodological variability currently limits inter-study comparability and hinders clinical translation. Future efforts should clarify the relative contributions and limitations of various quantification approaches, such as SUV metrics, RV/LV ratios, kinetic modeling, partial volume effect, and motion correction. Standardization of acquisition protocols, image processing pipelines, and implementation of multi-tracer studies are critical to fully realize the potential of PET imaging in PH. Moreover, future research should emphasize biological validation of tracer uptake in relation to right ventricular maladaptive remodeling and the integration of PET-derived metrics into multiparametric clinical models. Validation in large, multicenter cohorts will be essential to enhance diagnostic precision, guide therapeutic decisions, and ultimately enhance patient outcomes.

To overcome these limitations, a clear and prioritized research agenda must focus on the biological validation of tracer uptake in large, multicenter cohorts. However, without established guidelines and given the stated methodological issues, recommending PET for routine early detection or for guiding therapeutic interventions in PH is not yet supported by the presented evidence.

## Authors’ contributions

Pinto, Salvi de Souza, Marques da Silva, wrote the manuscript; Pinto, Salvi de Souza, performed the research; Pinto, Salvi de Souza, analyzed the data, Pinto, Salvi de Souza, Marques da Silva, Moriguchi Jeckel, Bodanese, Buchpiguel, and Souza review and editing; Marques da Silva designed and supervised the research.

## Funding

This study was financed in part by the Coordenação de Aperfeiçoamento de Pessoal de Nivel Superior (CAPES), Brasil – Finance Code 001.

## Data availability statement

The datasets generated and/or analyzed during the current study are available from the corresponding author upon reasonable request.

## Declaration of competing interest

The authors declare no conflicts of interest.

## Appendix Supplementary materials

Image, application 1

## References

[1] Galiè N., Humbert M., Vachiery J.L., Gibbs S., Lang I., Torbicki A. (2016). 2015 ESC/ERS Guidelines for the diagnosis and treatment of pulmonary hypertension. Eur Heart J.

[2] Koudstaal T., Boomars K.A., Kool M. (2020). Pulmonary arterial hypertension and chronic thromboembolic pulmonary hypertension: an immunological perspective. J Clin Med.

[3] Simonneau G., Montani D., Celermajer D.S., Denton C.P., Gatzoulis M.A., Krowka M. (2019). Haemodynamic definitions and updated clinical classification of pulmonary hypertension. Eur Respir J.

[4] Hoeper M.M., Humbert M., Souza R., Idrees M., Kawut S.M., Sliwa-Hahnle K. (2016). A global view of pulmonary hypertension. Lancet Respir Med.

[5] Bazan I.S., Fares WH. (2015). Pulmonary hypertension: diagnostic and therapeutic challenges. Ther Clin Risk Manag.

[6] Fuster V., Sanz J. (2007). [Pulmonary hypertension: new insights from techniques imaging]. Rev Esp Cardiol.

[7] Tatebe S., Fukumoto Y., Oikawa-Wakayama M., Sugimura K., Satoh K., Miura Y. (2014). Enhanced [18F]fluorodeoxyglucose accumulation in the right ventricular free wall predicts long-term prognosis of patients with pulmonary hypertension: a preliminary observational study. Eur Heart J Cardiovasc Imaging.

[8] Ahmad A., Zou Y., Zhang P., Li L., Wang X., Wang Y. (2024). Non-invasive imaging techniques for early diagnosis of bilateral cardiac dysfunction in pulmonary hypertension: current crests, future peaks. Front Cardiovasc Med.

[9] Vonk Noordegraaf A., Westerhof B.E., Westerhof N. (2017). The relationship between the right ventricle and its load in pulmonary hypertension. J Am Coll Cardiol.

[10] Alenezi F., Covington T.A., Mukherjee M., Mathai S.C., Yu P.B., Rajagopal S. (2022). Novel approaches to imaging the pulmonary vasculature and right heart. Circ Res.

[11] Sharma M., Burns A.T., Yap K., Prior DL. (2021). The role of imaging in pulmonary hypertension. Cardiovasc Diagn Ther.

[12] Hur D.J., Sugeng L. (2019). Non-invasive multimodality cardiovascular imaging of the right heart and pulmonary circulation in pulmonary hypertension. Front Cardiovasc Med.

[13] Hahn R.T., Lerakis S., Delgado V., Addetia K., Burkhoff D., Muraru D. (2023). Multimodality imaging of right heart function. J Am Coll Cardiol.

[14] Alabed S., Shahin Y., Garg P., Alandejani F., Johns C.S., Lewis R.A. (2021). Cardiac-MRI predicts clinical worsening and mortality in pulmonary arterial hypertension. JACC Cardiovasc Imaging.

[15] de Souza MT, da Silva MD, de Carvalho R (2010). Integrative review: what is it? How to do it?. Einstein (São Paulo).

[16] Tricco A.C., Lillie E., Zarin W., O’Brien K.K., Colquhoun H., Levac D. (2018). PRISMA extension for scoping reviews (PRISMA-ScR): checklist and explanation. Ann Intern Med.

[17] Johnson N., Phillips M. (2018). Rayyan for systematic reviews. J Electron Resour Libr.

[18] Kluge R., Barthel H., Pankau H., Seese A., Schauer J., Wirtz H. (2005). Different mechanisms for changes in glucose uptake of the right and left ventricular myocardium in pulmonary hypertension. J Nucl Med.

[19] Oikawa M., Kagaya Y., Otani H., Sakuma M., Demachi J., Suzuki J. (2005). Increased [18F]Fluorodeoxyglucose accumulation in right ventricular free wall in patients with pulmonary hypertension and the effect of Epoprostenol. J Am Coll Cardiol.

[20] Wong Y.Y., Raijmakers P., van Campen J., van der Laarse W.J., Knaapen P., Lubberink M. (2013). 11C-Acetate clearance as an index of oxygen consumption of the right myocardium in idiopathic pulmonary arterial hypertension: a validation study using 15O-labeled tracers and PET. J Nucl Med.

[21] Hagan G., Southwood M., Treacy C., Ross R.M., Soon E., Coulson J. (2011). 18 FDG PET imaging can quantify increased cellular metabolism in pulmonary arterial hypertension: a proof-of-principle study. Pulm Circ.

[22] Yoshinaga K., Ohira H., Tsujino I., Oyama-Manabe N., Mielniczuk L., Beanlands R.S.B. (2014). Attenuated right ventricular energetics evaluated using 11C-acetate PET in patients with pulmonary hypertension. Eur J Nucl Med Mol Imaging.

[23] Fang W., Zhao L., Xiong C., Ni X., He Z., He J. (2012). Comparison of 18F-FDG uptake by right ventricular myocardium in idiopathic pulmonary arterial hypertension and pulmonary arterial hypertension associated with congenital heart disease. Pulm Circ.

[24] Wang L., Zhang Y., Yan C., He J., Xiong C., Zhao S. (2013). Evaluation of right ventricular volume and ejection fraction by gated 18F-FDG PET in patients with pulmonary hypertension: comparison with cardiac MRI and CT. J Nucl Cardiol.

[25] Lundgrin E.L., Park M.M., Sharp J., Tang W.H.W., Thomas J.D., Asosingh K. (2013). Fasting 2-Deoxy-2-[18 F]fluoro- d -glucose positron emission tomography to detect metabolic changes in pulmonary arterial hypertension hearts over 1 year. Ann Am Thorac Soc.

[26] Can M.M., Kaymaz C., Tanboga I.H., Tokgoz H.C., Canpolat N., Turkyilmaz E. (2011). Increased right ventricular glucose metabolism in patients with pulmonary arterial hypertension. Clin Nucl Med.

[27] Bokhari S., Raina A., Berman Rosenweig E., Schulze P.C., Bokhari J., Einstein A.J. (2011). PET imaging may provide a novel biomarker and understanding of right ventricular dysfunction in patients with idiopathic pulmonary arterial hypertension. Circ Cardiovasc Imaging.

[28] Wong Y.Y., Westerhof N., Ruiter G., Lubberink M., Raijmakers P., Knaapen P. (2011). Systolic pulmonary artery pressure and heart rate are main determinants of oxygen consumption in the right ventricular myocardium of patients with idiopathic pulmonary arterial hypertension. Eur J Heart Fail.

[29] Wong Y.Y., Ruiter G., Lubberink M., Raijmakers P.G., Knaapen P., Marcus J.T. (2011). Right ventricular failure in idiopathic pulmonary arterial hypertension is associated with inefficient myocardial oxygen utilization. Circ Heart Fail.

[30] Wong Y.Y., Raijmakers P.G., Knaapen P., Lubberink M., Ruiter G., Marcus J.T. (2011). Supine-exercise-induced oxygen supply to the right myocardium is attenuated in patients with severe idiopathic pulmonary arterial hypertension. Heart.

[31] Yang T., Wang L., Xiong C.M., He J.G., Zhang Y., Gu Q. (2014). The ratio of 18F-FDG activity uptake between the right and left ventricle in patients with pulmonary hypertension correlates with the right ventricular function. Clin Nucl Med.

[32] Rijnierse M.T., Groeneveldt J.A., van Campen JSJA, de Boer K., van der Bruggen C.E.E., Harms H.J. (2020). Bisoprolol therapy does not reduce right ventricular sympathetic activity in pulmonary arterial hypertension patients. Pulm Circ.

[33] Oguz M., Kivrak T., Sunbul M., Dede F., Yildizeli B., Mutlu B. (2019). Diagnostic modality for evaluation of right ventricle in chronic thromboembolic pulmonary hypertension patients. Int J Cardiovasc Acad.

[34] Ahmadi A., Thornhill R.E., Pena E., Renaud J.M., Promislow S., Chandy G. (2018). Effects of riociguat on right ventricular remodelling in chronic thromboembolic pulmonary hypertension patients: a prospective study. Can J Cardiol.

[35] Wang L., Li W., Yang Y., Wu W., Cai Q., Ma X. (2016). Quantitative assessment of right ventricular glucose metabolism in idiopathic pulmonary arterial hypertension patients: a longitudinal study. Eur Heart J Cardiovasc Imaging.

[36] Wang L., Zhou W., Liang Y., Yang Y., Garcia E.V., Chen J. (2017). Right ventricular dyssynchrony in pulmonary hypertension: phase analysis using FDG-PET imaging. J Nucl Cardiol.

[37] Sakao S., Daimon M., Voelkel N.F., Miyauchi H., Jujo T., Sugiura T. (2016). Right ventricular sugars and fats in chronic thromboembolic pulmonary hypertension. Int J Cardiol.

[38] Ohira H., DeKemp R., Pena E., Davies R.A., Stewart D.J., Chandy G. (2016). Shifts in myocardial fatty acid and glucose metabolism in pulmonary arterial hypertension: a potential mechanism for a maladaptive right ventricular response. Eur Heart J Cardiovasc Imaging.

[39] Frille A., Steinhoff K.G., Hesse S., Grachtrup S., Wald A., Wirtz H. (2016). Thoracic [18F]fluorodeoxyglucose uptake measured by positron emission tomography/computed tomography in pulmonary hypertension. Med (Baltim).

[40] Li W., Wang L., Xiong C.M., Yang T., Zhang Y., Gu Q. (2015). The prognostic value of 18F-FDG uptake ratio between the right and left ventricles in idiopathic pulmonary arterial hypertension. Clin Nucl Med.

[41] Saygin D., Highland K.B., Farha S., Park M., Sharp J., Roach E.C. (2017). Metabolic and functional evaluation of the heart and lungs in pulmonary hypertension by gated 2-[18F]-Fluoro-2-deoxy-D-glucose positron emission tomography. Pulm Circ.

[42] Sumer C., Okumus G., Isik E.G., Turkmen C., Bilge A.K., Inanc M. (2024). 18F-fluorodeoxyglucose uptake by positron emission tomography in patients with IPAH and CTEPH. Pulm Circ.

[43] Gu Y., Han K., Zhang Z., Zhao Z., Yan C., Wang L. (2023). 68Ga-FAPI PET/CT for molecular assessment of fibroblast activation in right heart in pulmonary arterial hypertension: a single-center, pilot study. J Nucl Cardiol.

[44] Barton G.P., Corrado P.A., Francois C.J., Runo J.R., Chesler N.C., McMillan A.B. (2022). Development of a PET/MRI exercise stress test for determining cardiac glucose dependence in pulmonary arterial hypertension. Pulm Circ.

[45] Han Q.J., Forfia P., Vaidya A., Ramani G., deKemp R.A., Mach R.H. (2023). Effects of ranolazine on right ventricular function, fluid dynamics, and metabolism in patients with precapillary pulmonary hypertension: insights from a longitudinal, randomized, double-blinded, placebo controlled, multicenter study. Front Cardiovasc Med.

[46] Chen B.X., Xing H.Q., Gong J.N., Guo X.J., Xi X.Y., Yang Y.H. (2022). Imaging of cardiac fibroblast activation in patients with chronic thromboembolic pulmonary hypertension. Eur J Nucl Med Mol Imaging.

[47] Farha S., Comhair S., Hou Y., Park M.M., Sharp J., Peterson L. (2021). Metabolic endophenotype associated with right ventricular glucose uptake in pulmonary hypertension. Pulm Circ.

[48] Kazimierczyk R., Malek L.A., Szumowski P., Nekolla S.G., Blaszczak P., Jurgilewicz D. (2021). Multimodal assessment of right ventricle overload-metabolic and clinical consequences in pulmonary arterial hypertension. J Cardiovasc Magn Reson.

[49] Kazimierczyk R., Szumowski P., Nekolla S., Małek Ł, Błaszczak P., Hładuński M. (2022). Platelet sTWEAK and plasma IL-6 are associated with 18F-fluorodeoxyglucose uptake in right ventricles of patients with pulmonary arterial hypertension: a pilot study. Adv Clin Exp Med.

[50] Kazimierczyk R., Szumowski P., Nekolla S.G., Blaszczak P., Malek L.A., Milosz-Wieczorek B. (2021). Prognostic role of PET/MRI hybrid imaging in patients with pulmonary arterial hypertension. Heart.

[51] Kazimierczyk R., Szumowski P., Nekolla S.G., Malek L.A., Blaszczak P., Hladunski M. (2023). The impact of specific pulmonary arterial hypertension therapy on cardiac fluorodeoxyglucose distribution in PET/MRI hybrid imaging-follow-up study. EJNMMI Res.

[52] Ryan J.J., Archer SL. (2014). The right ventricle in pulmonary arterial hypertension. Circ Res.

[53] Schirone L., Forte M., Palmerio S., Yee D., Nocella C., Angelini F. (2017). A review of the molecular mechanisms underlying the development and progression of cardiac remodeling. Oxid Med Cell Longev.

[54] Bom M.J., van Diemen P.A., Driessen R.S., Everaars H., Schumacher S.P., Wijmenga J.T. (2020). Prognostic value of [15O]H2O positron emission tomography-derived global and regional myocardial perfusion. Eur Heart J Cardiovasc Imaging.

[55] Kudomi N., Kalliokoski K.K., Oikonen V.J., Han C., Kemppainen J., Sipilä H.T. (2019). Myocardial blood flow and metabolic rate of oxygen measurement in the right and left ventricles at rest and during exercise using 15O-labeled compounds and PET. Front Physiol.

[56] Koop A.M.C., Bossers G.P.L., Ploegstra M.J., Hagdorn Q.A.J., Berger R.M.F., Silljé H.H.W. (2019). Metabolic remodeling in the pressure-loaded right ventricle: shifts in glucose and fatty acid metabolism—a systematic review and meta-analysis. J Am Heart Assoc.

[57] Sakamaki F., Satoh T., Nagaya N., Kyotani S., Oya H., Nakanishi N. (2000). Correlation between severity of pulmonary arterial hypertension and 123I-metaiodobenzylguanidine left ventricular imaging. J Nucl Med.

[58] Wang L., Zhang Y., Yan C., He J., Xiong C., Zhao S. (2013). Evaluation of right ventricular volume and ejection fraction by gated 18F-FDG PET in patients with pulmonary hypertension: comparison with cardiac MRI and CT. J Nucl Cardiol.

[59] Kim J.Y., Suh Y.J., Han K., Kim Y.J., Choi BW. (2020). Cardiac CT for measurement of right ventricular volume and function in comparison with cardiac MRI: a meta-analysis. Korean J Radiol.

[60] Maffei E., Messalli G., Martini C., Nieman K., Catalano O., Rossi A. (2012). Left and right ventricle assessment with Cardiac CT: validation study vs. Cardiac MR. Eur Radiol.

[61] De Luca G.M.R., Habraken JBA. (2022). Method to determine the statistical technical variability of SUV metrics. EJNMMI Phys.

[62] Kinahan P.E., Fletcher JW. (2010). Positron emission tomography-computed tomography standardized uptake values in clinical practice and assessing response to therapy. Semin Ultrasound CT MR.

[63] Mukaka MM. (2012). Statistics corner: a guide to appropriate use of correlation coefficient in medical research. Malawi Med J.

[64] Zhang H., Li M., Hu C.J., Stenmark KR. (2024). Fibroblasts in pulmonary hypertension: roles and molecular mechanisms. Cells.

[65] Adams M.C., Turkington T.G., Wilson J.M., Wong TZ. (2010). A systematic review of the factors affecting accuracy of SUV measurements. AJR Am J Roentgenol.

[66] Vanderhoek M., Perlman S.B., Jeraj R. (2013). Impact of different standardized uptake value measures on PET-based quantification of treatment response. J Nucl Med.

[67] Bettinardi V., Castiglioni I., De Bernardi E., Gilardi MC. (2014). PET quantification: strategies for partial volume correction. Clin Transl Imaging.

[68] Cysouw M.C.F., Golla S.V.S., Frings V., Smit E.F., Hoekstra O.S., Kramer G.M. (2019). Partial-volume correction in dynamic PET-CT: effect on tumor kinetic parameter estimation and validation of simplified metrics. EJNMMI Res.

[69] Slart RHJA, Martinez-Lucio T.S., Boersma H.H., Borra R.H., Cornelissen B., Dierckx RAJO (2024). [15O]H_2_O PET: potential or Essential for Molecular Imaging?. Semin Nucl Med.

[70] Gu Y., Han K., Zhang Z., Zhao Z., Yan C., Wang L. (2023). 68Ga-FAPI PET/CT for molecular assessment of fibroblast activation in right heart in pulmonary arterial hypertension: a single-center, pilot study. J Nucl Cardiol.

[71] Petibon Y., Sun T., Han P.K., Ma C., El Fakhri G, Ouyang J. (2019). MR-based cardiac and respiratory motion correction of PET: application to static and dynamic cardiac 18F-FDG imaging. Phys Med Biol.

[72] Petibon Y., Guehl N.J., Reese T.G., Ebrahimi B., Normandin M.D., Shoup T.M. (2017). Impact of motion and partial volume effects correction on PET myocardial perfusion imaging using simultaneous PET-MR. Phys Med Biol.

[73] Humbert M., Kovacs G., Hoeper M.M., Badagliacca R., Berger R.M.F., Brida M. (2022). 2022 ESC/ERS Guidelines for the diagnosis and treatment of pulmonary hypertension. Eur Heart J.

[74] Reiter U., Reiter C., Kräuter C., Fuchsjäger M., Reiter G. (2018). Cardiac magnetic resonance T1 mapping. Part 2: diagnostic potential and applications. Eur J Radiol.

