# Supplementary material for: Role of PET imaging in evaluating right ventricular function and prognosis in pulmonary hypertension
Source: Clinics (Sao Paulo). 2026 Sep 7;81:101101. doi: 10.1016/j.clinsp.2026.101101 (PMC13578966; doi:10.1016/j.clinsp.2026.101101)
Supplement: Supplementary file 1 [file mmc1.docx]

CLINICS-D-26-00004

**Supplementary Material**

**Supplementary Table S1** Search strategies for each database.

|  | **P (Population)** | **C (Concept)** | **C (Context)** | **Search result** |
| --- | --- | --- | --- | --- |
| **EXTRACTION** |  |  |  |  |
| **CONVERSION** (Controlled vocabulary) | Subjects with pulmonary hypertension | PET imaging | Quantification | July 1^st^, 2024 |
| **MEDLINE (PubMed),** (Using indexed descriptors Medical Subject Headings (MeSH)) | ("PET" OR "Positron Emission Tomography" OR "Positron-Emission Tomography" OR "Positron Emission Tomography" OR "Positron-Emission Tomography Imaging" OR "Imaging, Positron-Emission Tomography" OR "Positron Emission Tomography Imaging" OR "Positron-Emission Tomography Imagings" OR "Tomography Imaging, Positron-Emission" OR "Tomography, Positron-Emission" OR "Tomography, Positron Emission" OR "PET Scan" OR "PET Scans" OR "Scan, PET" OR "PET Imaging" OR "Imaging, PET" OR "PET Imagings" AND ("right ventricle" OR "right ventricular" OR "right heart" OR "Right Ventricle" OR "Right Ventricles" OR "Ventricle, Right" OR "Ventricles, Right") AND ("Pulmonary Arterial Hypertension" OR "Heritable Pulmonary Arterial Hypertension" OR "Hypertension, Idiopathic Pulmonary" OR "Hypertension, Primary Pulmonary" OR "Idiopathic Pulmonary Arterial Hypertension" OR "Idiopathic Pulmonary Hypertension" OR "Primary Pulmonary Hypertension" OR "Pulmonary Hypertension, Idiopathic") | | | 56 |
| **Web of Science** | (Using indexed descriptors Medical Subject Headings (MeSH) | | | 88 |
| **Scopus** | (Using indexed descriptors Medical Subject Headings (MeSH) | | | 65 |
| **Embase** (Using indexed descriptors Emtree) | ('p.e.t.' OR 'PET scan' OR 'PET scanning' OR 'positron emission tomographic scan' OR 'positron emission tomographic scanning' OR 'positron tomography' OR 'positron-emission tomography' OR 'tomography, positron' OR 'positron emission tomography') AND ('heart ventricle, right' OR 'right heart ventricle' OR 'right ventricle' OR 'ventricle, right' OR 'heart right ventricle') AND ('essential pulmonary hypertension' OR 'familial primary pulmonary hypertension' OR 'hypertension, pulmonary' OR 'hypertensive pulmonary vascular disease' OR 'idiopathic pulmonary arterial hypertension' OR 'primary pulmonary hypertension' OR 'pulmonary arterial hypertension' OR 'pulmonary artery hypertension' OR 'pulmonary fixed hypertension' OR 'pulmonary hypertensive disease' OR 'pulmonary hypertensive diseases' OR 'pulmonary hypertensive disorder' OR 'pulmonary hypertensive disorders' OR 'pulmonary hypertension') | | | 260 |
| **Total** | | | | 469 |

**Supplementary Table S2** Description of the population and quantification parameters used in the studies.

| **Population** | **Parameters quantify** | **Study** | **n** |
| --- | --- | --- | --- |
| **Patients and Controls** | SUV, SUV_mean_, SUVR, TBR, RV/LV, RVSP, RAP, NT-pro BNP, PVR, RVPSS: | 21; 22; 26; 41; 42; 47; 48; 50 | 8 |
| **Patients** | SUV, SUV_mean_, SUV_max_, cSUV, SUVR, MVO_2_, Ki, OEF, MGU, MGUR, TBR, MBF, RV/LV, RVEDV, RVESV, RVBW, RVSD; VEF FBV. | 7, 18–20, 23–25, 27–40, 43–46, 49, 51 | 27 |

cSUV, Corrected SUV; FBV, Fractional Blood Volume; Ki, Influx rate constant; MBF, Myocardial Blood Flow; MGU, Myocardial Glucose Uptake; MGUR, MGU (RV/LV) Myocardial Glucose Uptake Ratio; MVO_2_, Myocardial Oxygen consumption; NT-pro BNP; NT-proBNP, N-terminal pro-B-type Natriuretic Peptide; OEF, O_2_ Extraction Fraction; PVR, Pulmonary Vascular Resistance; RAP, Right Atrial Pressure; RV/LV, Ratio Right Ventricle to Left Ventricle; RVBW, Histogram Bandwidth; RVEDV, Right Ventricular End-Diastolic Volume; RVEF, Right Ventricular Ejection Fraction; RVESV, Right Ventricular End-Systolic Volume; RVPSS, RV longitudinal global Peak Systolic Strain; RVSD, Right Ventricular Phase Standard Deviation; RVSP, Right Ventricular Systolic Pressure; SUV, Standardized Uptake Value; SUVR, SUV (RV/LV) Ratio; TBR, Tissue-to-Blood Ratio.

**Supplementary Table S3** Quality assessment of included studies using the Newcastle-Ottawa Scale (NOS).

| **Study authors** | **Selection (max. 4)** | **Comparability (max. 2)** | **Outcome/Exposure (max. 3)** | **Total score (max. 9)** |
| --- | --- | --- | --- | --- |
| Sumer et al., 2024 [42] | **4**  Representativeness of sample, selection of controls (oncology), validated exposure, ascertainment of PH | **1**  Adjustment for demographics only | **3**  Validated tools for PET, blinded outcome assessment, adequate follow-up | **8** |
| Han et al., 2023 [45] | **4**  Representativeness of sample, exposure definition, ascertainment, independent controls | **1**  Adjustment for age | **3**  Validated measurement tools, appropriate follow-up, statistical clarity | **8** |
| Li et al., 2015 [40] | **3**  Validated exposure definition, ascertainment, representativeness (no matched healthy controls) | **1**  Adjustment for baseline demographics | **3**  Validated tools for PET quantification, blinded assessment, robust follow-up | **7** |
| Kluge et al., 2005 [18] | **3**  Consecutive patients, validated exposure, ascertainment (unmatched controls) | **1**  Adjustment for age | **3**  Validated PET tools, adequate follow-up, blinded outcome | **7** |
| Tatebe et al., 2014 [7] | **3**  Representativeness of sample, exposure definition, ascertainment | **1**  Adjustment for age and sex only | **3**  Validated outcome measurement, appropriate follow-up, blinded assessment | **7** |
| Farha et al., 2021 [47] | **3**  Population sample, validated exposure definition, ascertainment | **1**  Adjustment for basic demographics | **3**  Validated PET tools, adequate follow-up, statistical robustness | **7** |
| Kazimierczyk et al., 2021a [48] | **3**  Representativeness of sample, exposure definition, ascertainment | **1**  Adjustment for clinical variables | **3**  Validated outcome measurement, large cohort follow-up | **7** |
| Kazimierczyk et al., 2021b [50] | **3**  Consecutive patients, validated exposure, ascertainment | **1**  Adjustment for age | **3**  Validated tools for PET, blinded outcome assessment | **7** |
| Hagan et al., 2011 [21] | **3**  Validated exposure definition, representativeness, ascertainment | **1**  Adjustment for demographics only | **3**  Validated outcome measurement, appropriate follow-up | **7** |
| Yoshinaga et al., 2014 [22] | **3**  Representativeness of sample, exposure definition, ascertainment | **1**  Adjustment for basic clinical factors | **3**  Validated kinetic modeling tools, adequate follow-up | **7** |
| Rijnierse et al., 2020 [32] | **3**  Well-defined criteria, validated exposure, ascertainment | **1**  Adjustment for demographics only | **3**  Validated tools, appropriate follow-up, blinded assessment | **7** |
| Can et al., 2011 [26] | **3**  Validated exposure definition, ascertainment, representativeness | **0**  No adjustment for confounding baseline therapies | **3**  Validated tools for PET, adequate follow-up | **6** |
| Chen et al., 2022 [46] | **3**  Consecutive patients, validated exposure, ascertainment | **0**  No adjustment for comorbidities or PAH drugs | **3**  Validated outcome measurement, adequate follow-up | **6** |
| Oikawa et al., 2005 [19] | **3**  Validated exposure definition, ascertainment, representativeness | **0**  No adjustment for baseline demographics | **3**  Validated tools for PET quantification, appropriate follow-up | **6** |
| Wang et al., 2013 [24] | **3**  Representativeness of sample, exposure definition, ascertainment | **0**  No adjustment for sex mismatch | **3**  Validated outcome measurement, blinded assessment | **6** |
| Lundgrin et al., 2013 [25] | **3**  Validated exposure definition, ascertainment, representativeness | **0**  No adjustment for extreme size imbalance | **3**  Validated tools for PET, appropriate follow-up | **6** |
| Saygin et al., 2017 [41] | **3**  Consecutive patients, validated exposure, ascertainment | **0**  No adjustment for confounding baseline therapies | **3**  Validated outcome measurement, adequate follow-up | **6** |
| Kazimierczyk et al., 2022 [49] | **3**  Validated exposure definition, ascertainment, representativeness | **0**  No adjustment for prior PAH therapies | **3**  Validated PET kinetic modeling, adequate follow-up | **6** |
| Wang et al., 2016 [35] | **3**  Representativeness of sample, exposure definition, ascertainment | **0**  Severe age mismatch not adjusted | **3**  Validated tools for PET, blinded assessment | **6** |
| Wang et al., 2017 [36] | **3**  Validated exposure definition, ascertainment, representativeness | **0**  No adjustment for baseline therapies | **3**  Validated outcome measurement, appropriate follow-up | **6** |
| Sakao et al., 2016 [37] | **3**  Consecutive patients, validated exposure, ascertainment | **0**  No adjustment for dietary confounders | **3**  Validated tools for PET quantification, adequate follow-up | **6** |
| Ohira et al., 2016 [38] | **3**  Representativeness of sample, exposure definition, ascertainment | **0**  No adjustment for baseline comorbidities | **3**  Validated outcome measurement, appropriate follow-up | **6** |
| Wong et al., 2011a [28] | **3**  Validated exposure definition, ascertainment, representativeness | **0**  No adjustment for basic demographics | **3**  Validated dynamic PET tools, adequate follow-up | **6** |
| Kazimierczyk et al., 2023 [51] | **3**  Consecutive patients, validated exposure, ascertainment | **0**  No adjustment for disease progression confounders | **3**  Validated outcome measurement, appropriate follow-up | **6** |
| Fang et al., 2012 [23] | **3**  Validated exposure definition, ascertainment, representativeness | **0**  No adjustment for baseline therapies | **3**  Validated tools for PET, adequate follow-up | **6** |
| Wong et al., 2013 [20] | **2**  Validated exposure, ascertainment (Total lack of healthy controls) | **0**  No adjustment for comorbidities or therapies | **3**  Validated kinetic modeling tools, appropriate follow-up | **5** |
| Gu et al., 2023 [43] | **2**  Validated exposure, ascertainment (Small pilot sample, no controls) | **0**  No adjustment for baseline confounders | **3**  Validated tools for PET, blinded assessment | **5** |
| Barton et al., 2022 [44] | **2**  Ascertainment, validated exposure (Extreme selection bias) | **0**  No adjustment for demographics or PAH drugs | **3**  Validated outcome measurement, appropriate follow-up | **5** |
| Oguz et al., 2019 [33] | **2**  Validated exposure, ascertainment (No independent controls) | **0**  No statistical adjustment for prior therapies | **3**  Validated tools for PET, adequate follow-up | **5** |
| Ahmadi et al., 2018 [34] | **2**  Validated exposure, ascertainment (Extremely small sample, no controls) | **0**  No adjustment for confounding baseline therapies | **3**  Validated kinetic tools, appropriate follow-up | **5** |
| Bokhari et al., 2010 [27] | **2**  Ascertainment, validated exposure (Total absence of healthy controls) | **0**  No adjustment for basic demographics | **3**  Validated tools for PET quantification, adequate follow-up | **5** |
| Wong et al., 2011b [29] | **2**  Validated exposure, ascertainment (Extreme selection bias, no controls) | **0**  No adjustment for hemodynamics | **3**  Validated dynamic PET tools, adequate follow-up | **5** |
| Wong et al., 2011c [30] | **2**  Validated exposure, ascertainment (Small sample size, no controls) | **0**  No adjustment for baseline therapies | **3**  Validated dynamic PET tools, appropriate follow-up | **5** |
| Yang et al., 2014 [31] | **2**  Validated exposure, ascertainment (Internal comparisons only) | **0**  No adjustment for baseline confounders | **3**  Validated outcome measurement, blinded assessment | **5** |
| Frille et al., 2016 [39] | **2**  Validated exposure, ascertainment (High selection bias, end-stage only) | **0**  No adjustment for prior PAH therapies | **3**  Validated tools for PET, adequate follow-up | **5** |

**Supplementary Table S4** Moderate correlations (*r*≥0.5 and *r*<0.7) between PET parameters and clinical measures of PH severity from the reviewed articles.

| **Study** | **Tracer** | **Group** | **Simple Size (n)** | **PET parameter** | **Physiological parameters** | **Correlation (*r*)** |
| --- | --- | --- | --- | --- | --- | --- |
| Wong et al. 2011 [30] | [^15^O]H_2_O, [^15^O]O_2_, [^15^O]CO | PH due to left heart disease and CTEPH | n=18 | MVO_2_ | Stroke volume | 0.57 |
| Wong et al. 2013 [20] | [^15^O]CO | IPAH | n=26 | K_mono_ | MVO_2_ | 0.64 |
|  |  |  |  |  | RPP | 0.64 |
| Wong et al. 2013 [20] | [^15^O]CO | IPAH | n=26 | k_2_ | MVO_2_ | 0.67 |
|  |  |  |  |  | RPP | 0.69 |
| Hagan et al. 2011 [21] | [^18^F]FDG | IPAH and CTEPH | n=14 | SUV | NT-ProBNP | 0.55 |
| Gu et al. 2023 [43] | [^68^Ga]FAPI | PAH | n=16 | SUV_max_ | TPR | 0.53 |
|  |  |  |  |  | NT-ProBNP | 0.61 |
| Yoshinaga et al. 2014 [22] | [^11^C]acetate | PH due to left heart disease and CTEPH | n=27 patients; n=9 controls | K_mono_ | PVR | 0.58 |
| Rijnierse et al. 2020 [32] | [^11^C]HED | IPAH | n=13 | RI | Norepinephrine | 0.66 |
|  |  |  |  |  | RVEF | 0.66 |
|  |  |  |  |  | Wall tension | 0.69 |
|  |  |  |  |  | HRRV | 0.68 |
|  |  |  |  |  | CO | 0.67 |
|  |  |  |  |  | PVR | 0.60 |
|  |  |  |  |  | RAP | 0.57 |
| Fang et al. 2012 [23] | [^18^F]FDG | IPHA | n=24 | SUVR | mPAP | 0.56 |
|  |  |  |  |  | REVDP | 0.61 |
|  |  |  |  |  | PVR | 0.57 |
|  |  |  |  |  | CI | - 0.503 |
| Fang et al. 2012 [23] | [^18^F]FDG | CHD – PAH | n=43 | SUVR | PVR | 0.52 |
|  |  |  |  |  | 6MWT | -0.55 |
| Wang et al. 2013 [24] | [^18^F]FDG | PH | n=23 | SUV | RVEF-CMR | 0.54 |
| Wang et al. 2016 [35] | [^18^F]FDG | IPAH | n=27 patients; n=21 controls | MGUR | mPAP | 0.56 |
|  |  |  |  |  | RVEF | 0.55 |
|  |  |  |  |  | RV Tei index | 0.55 |
| Wang et al. 2017 [36] | [^18^F]FDG | PH | n=44 | SUVR | RVEF-CMR | 0.52 |
| Lundgrin et al. 2013 [25] | [^18^F]FDG | PAH | n=14 patients; n=6 controls | TBR | RV end systolic diameter; Systolic | Rho=0.6 |
| Chen et al. 2022 [46] | [^68^Ga]FAPI-04 | CTEPH | n=13 | TBR | RV FAC | - 0.68 |
| Oikawa et al. 2005 [19] | [^18^F]FDG | PH | n=24 | SUV | PVR | 0.67 |
|  |  |  |  |  | RAP | 0.50 |
|  |  |  |  |  | PBNPL | 0.54 |
| Can et al. 2011 [26] | [^18^F]FDG | PAH | n=23 patients; n=16 controls | SUVR | BNP | 0.63 |
|  |  |  |  |  |  | 0.61 |
|  |  |  |  |  | St | -0.68 |
| Saygin et al. 2017 [41] | [^18^F]FDG | PH | n=30 patients; n=8 controls | SUVR | RAP | 0.50 |
|  |  |  |  |  | RVED areas | 0.60 |
|  |  |  |  |  | RVFS | 0.50 |
| Kazimierczyk, Malek, et al. 2021 [48] | [^18^F]FDG | PH due to left heart disease and PH due to lung disease | n=27 | SUVR | RV GLS/PASP | 0.67 |
|  |  |  |  |  | RV GLS/PVR | 0.55 |
|  |  |  |  |  | RV GLS*PAC | 0.69 |
| Kazimierczyk, Szumowski, et al. 2021 [50] | [^18^F]FDG | PAH | n=26 patients; n=12 controls | SUVR | RVEF | -0.52 |
|  |  |  |  |  | RV thickness | 0.51 |
| Kazimierczyk et al. 2022 [49] | [^18^F]FDG | PAH | n=18 | SUVR | Platelet sTWEAK levels | -0.57 |
|  |  |  |  |  | Plasma IL-6 a | 0.50 |
|  |  |  |  |  | RVEF | -0.52 |
|  |  |  |  |  | PVR | 0.62 |
| Kazimierczyk et al. 2023 [51] | [^18^F]FDG | PAH | n=20 | SUVR | BNP | 0.58 |
|  |  |  |  |  | RVEF | -0.60 |
| Bokhari et al. 2011 [27] | [^18^F]FDG | IPAH | n=16 | MGU free wall RV | mPAP | 0.66 |
| Sakao et al. 2016 [37] | [^18^F]FDG, and [^123^I]BMIPP | CTEPH | n=20 | SUV | mPAP | 0.50 |
|  |  |  |  |  | PVR | 0.57 |
|  |  |  |  |  | RV wall thickness | 0.62 |
| Ohira et al. 2016 [38] | [^18^F]FDG | PAH | n=17 | SUVR | mPAP | 0.68 |
|  |  |  |  |  | PVR | 0.69 |
|  |  |  |  |  | RVEF | -0.56 |
| Ohira et al. 2016 [38] | [^18^F]FTHA | PAH | n=17 | SUVR | RVEF | -0.62 |
| Ohira et al. 2016 [38] | [^18^F]FDG, and [^18^F]FTHA | PAH | n=17 |  | mPAP | 0.60 |
| Yang et al. 2014 [31] | [^18^F]FDG | IPAH | n=38 | fSUVr | PVR | 0.63 |
|  |  |  |  | fSUV_R/L_ |  | 0.58 |
|  |  |  |  | gSUV_R/L_ |  | 0.62 |

6MWD, 6-Minute Walk Distance; BNP, B-type Natriuretic Peptide; CI, Cardiac Index; CO, Cardiac Output; CTEPH, Chronic Thromboembolic Pulmonary Hypertension; fSUVR/L, SUV fasting; fSUVR/L, SUV glucose-Loading; FAC, Fractional Area Change; GLS, Global Peak Systolic Strain; HRRV, Heart Rate and RV; IPAH, Idiopathic Pulmonary Arterial Hypertension; MGRU, MGU (RV/LV) Myocardial Glucose Uptake Ratio; MGU, Myocardial Glucose Uptake; mPAP, Mean Pulmonary Artery Pressure; MVO_2_, Myocardial Oxygen consumption; NT-proBNP, N-terminal pro-B-type Natriuretic Peptide; O_2_ extraction fraction; PAC, Pulmonary Arterial Compliance: PAH, Pulmonary Arterial Hypertension; PASP, Systolic Pulmonary Artery Pressure; PH, Pulmonary Hypertension; PVR, Pulmonary Vascular Resistance; RAP, Right Atrial Pressure; RI, Retention Index; RPP, Rate-Pressure Product; RV/LV, Ratio Right Ventricle to left ventricle; RVED: RV end-diastolic; RVEDV: right ventricular end-diastolic Volume; RVEF, Right Ventricular Ejection Fraction; RVES, RV End-Systolic; St, Tricuspid annular systolic velocity; SUV, Standardized Uptake Value; SUVR, SUV (RV/LV) Ratio; TAPSE, Tricuspid Annular Plane Systolic Excursion; TBR, Tissue-to-Blood Ratio; TPR, Total Pulmonary Resistance.

**Supplementary Table S5** Low and negligible correlations (*r* < 0.5) between PET parameters and clinical measures of PH severity from the reviewed articles.

| **Study** | **Tracer** | **Group** | **Sample Size (n)** | **PET parameter** | **Physiological parameters** | **Correlation (*r*)** |
| --- | --- | --- | --- | --- | --- | --- |
| Yoshinaga et al. 2014 [22] | [^11^C]acetate | PH due to left heart disease and CTEPH | n = 27 patients | Kmono | mPAP | 0.46 |
|  |  |  | n = 9 controls |  | NT-proBNP | 0.26 |
| Fang et al. 2012 [23] | [^18^F]FDG | IPHA | n = 43 | SUVR | RAP | 0.47 |
| Oguz et al. 2019 [33] | [^18^F]FDG | CTEPH | n = 53 | SUVR | 6MWT | -0.46 |
|  |  |  |  |  | NT-ProBNP | 0.40 |
|  |  |  |  |  | TAPSE | -0.22 |
|  |  |  |  |  | MPI | -0.24 |
|  |  |  |  |  | RVS | -0.27 |
|  |  |  |  |  | RA area | 0.39 |
|  |  |  |  |  | LA area | -0.22 |
|  |  |  |  |  | RVEDD | 0.22 |
|  |  |  |  |  | LVEDD | - 0.47 |
|  |  |  |  |  | LVESD | -0.34 |
|  |  |  |  |  | sPAP | 0.43 |
|  |  |  |  |  | mPAP | 0.24 |
|  |  |  |  |  | DPAP | 0.33 |
|  |  |  |  |  | CO | 0.17 |
|  |  |  |  |  | CI | -0.13 |
|  |  |  |  |  | TG | 0.32 |
|  |  |  |  |  | DPG | 0.36 |
|  |  |  |  |  | PVR | 0.30 |
|  |  |  |  |  | SVR | 0.107 |
| Wang et al. 2013 [24] | [^18^F]FDG | PH | n = 23 | SUV | RVEF-CCT | 0.43 |
| Wang et al. 2016 [35] | [^18^F]FDG | IPAH | n = 27 patients; n = 21 controls | MGUR | sPAP | 0.43 |
|  |  |  |  |  | DPAP | 0.47 |
|  |  |  |  |  | PVR | 0.45 |
|  |  |  |  |  | TAPSE | 0.406 |
|  |  |  |  |  | RA area | 0.46 |
| Wang et al. 2017 [36] | [^18^F]FDG | PH | n = 44 | SUVR | RVEF-CCT | 0.43 |
| Can et al. 2011 [26] | [^18^F]FDG | PAH | n = 23 patients; n = 16 controls | SUVR | Tei index | 0.47 |
| Saygin et al. 2017 [41] | [^18^F]FDG | PH | n = 30 patients; n = 8 controls | SUVR | RA volume | 0.40 |
|  |  |  |  |  | RVES | 0.40 |
|  |  |  |  |  | RVSP | 0.30 |
|  |  |  |  |  | TAPSE | 0.40 |
| Kazimierczyk et al. 2023 [51] | [^18^F]FDG | PAH | n = 20 | SUVR | RV thickness | 0.41 |
| Sakao et al. 2016 [37] | [^18^F]FDG, and [^123^I]BMIPP | CTEPH | n = 20 | SUV | CI | 0.47 |
|  |  |  |  |  | BMIPP | 0.46 |
| Yang et al. 2014 [39] | [^18^F]FDG | IPAH | n = 38 | fSUVr | RVEF | -0.36 |
|  |  |  |  | gSUVr |  | -0.34 |
|  |  |  |  | fSUV_R/L_ |  | -0.34 |
|  |  |  |  | gSUV_R/L_ |  | -0.51 |
| Yang et al. 2014 [31] | [^18^F]FDG | IPAH | n = 38 | gSUVr | PVR | 0.40 |

6MWD, 6-Minute Walk Distance; BMIPP, 123I-β-Methyl Iodophenyl Pentadecanoic acid; CI, Cardiac Index; CO, Cardiac Output; CTEPH, Chronic Thromboembolic Pulmonary Hypertension; DPAP, Diastolic Pulmonary Artery Pressure; DPG, Diastolic Pulmonary Gradient; fSUVr, SUV fasting; gSUVr, SUV Glucose-loading; IPAH, Idiopathic Pulmonary Arterial Hypertension; LA, Left Atrium Area; LVEDD, Left Ventricle End-Diastolic Diameter; LVESD, Left Ventricle End Systolic Diameter; MGRU, MGU (RV/LV) Myocardial Glucose Uptake Ratio; MGU, Myocardial Glucose Uptake; mPAP, Mean Pulmonary Artery Pressure; NT-proBNP, N-terminal pro-B-type Natriuretic Peptide; PAH, Idiopathic Pulmonary Arterial Hypertension; PH, Pulmonary Hypertension; PVR, Pulmonary Vascular Resistance; RA, Right Atrium Area; RAP, Right Atrial Pressure; RVED, RV End-Diastolic; RVEF, Right Ventricular Ejection Fraction; RVES, RV End-Systolic; RVS, Tricuspid annulus tissue Doppler Systolic Velocity; RVSP, Right Ventricular Systolic Pressure; sPAP, Systolic Pulmonary Arterial Pressure; SUV, Standardized Uptake Value; SUVR, SUV (RV/LV) Ratio; SVR, Systemic Vascular Resistance; TAPSE, Tricuspid Annular Plane Systolic Excursion; TG, Transpulmonary gradient.
